# Strong and Thermo‐Switchable Gel Adhesion Based on UCST‐Type Phase Transition in Deep Eutectic Solvent

**DOI:** 10.1002/advs.202400938

**Published:** 2024-06-17

**Authors:** Huiyao Xu, Haocheng Li, Yan Zhang, Ying Guan, Yongjun Zhang

**Affiliations:** ^1^ Key Laboratory of Functional Polymer Materials and State Key Laboratory of Medicinal Chemical Biology Institute of Polymer Chemistry College of Chemistry Nankai University Tianjin 300071 China; ^2^ School of Pharmaceutical Sciences Tiangong University Tianjin 300387 China; ^3^ Cangzhou Institute of Tiangong University Cangzhou 061000 China

**Keywords:** deep eutectic solvent, gels, mechanical properties, reversible adhesion, thermosensitive

## Abstract

It remains a great challenge to achieve strong and reversible hydrogel adhesion. Hydrogel adhesives also suffer from poor environmental stability due to dehydration. To overcome these problems, here reversible adhesive gels are designed using a new switching mechanism and new solvent. For the first time, the study observes UCST (upper critical solution temperature)‐type thermosensitive behaviors of poly(benzyl acrylate) (PBnA) polymer and gel in menthol:thymol deep eutectic solvents (DESs). The temperature‐induced phase transition allows adjusting cohesive force, and hence adhesion strength of PBnA gels by temperature. To further improve the mechanical and adhesion properties, a peptide crosslinker is used to allow energy dissipation when deforming. The resulting eutectogel exhibits thermal reversible adhesion with a high switching ratio of 14.0. The adhesion strength at attachment state reaches 0.627 MPa, which is much higher than most reversible adhesive hydrogels reported before. The low vapor pressure of DES endows the gel excellent environmental stability. More importantly, the gel can be repeatedly switched between attachment and detachment states. The strong and reversible gel adhesive is successfully used to design soft gripper for the transport of heavy cargos and climbing robot capable of moving on vertical and inverted surface in a manner similar to gecko.

## Graphical abstract

Exploiting UCST‐type phase transition an adhesive eutectogel that can be repeatedly switched between attachment and detachment states, with high switching ratio (14.0) and high adhesion strength (0.627 MPa), is synthesized and successfully used to design soft gripper for the transport of heavy cargos and climbing robot capable of moving on vertical and inverted surface in a manner similar to gecko.

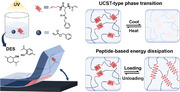

## 1 Introduction

Smooth switching between attachment and detachment states of gecko setae allows the animal to run with reckless abandon on wall and ceiling.^[^ ^1^ ^]^ The phenomenon amazed people since ancient times and inspired the development of reversible adhesives in recent years.^[^ ^2^ ^]^ Particularly reversible hydrogel adhesives have been developed.^[^ ^2^, ^3^ ^]^ These adhesives can be detached on demand by applying external stimuli such as temperature,^[^ ^4^, ^5^, ^6^ ^]^ pH,^[^ ^7^ ^]^ electricity^[^ ^8^ ^]^ and light,^[^ ^9^ ^]^ and found important applications in climbing robots,^[^ ^5^, ^8^, ^9^ ^]^ gripers,^[^ ^6^, ^10^ ^]^ hemostasis,^[^ ^11^ ^]^ tissue sealant^[^ ^12^ ^]^ and wearable iontronics.^[^ ^13^ ^]^

Various methods were proposed to design reversible hydrogel adhesives. A straightforward strategy to achieve reversible adhesion is to adjust the interfacial adhesion force between the gel and the substrate. For example, by exposing and shielding the adhesive groups at the interface, such as catechol groups, hydrogels can be tuned from adhesive to non‐adhesive.^[^ ^5^, ^6^, ^8^, ^9^, ^10^ ^]^ However, the adhesion strength at attachment state of this type of reversible gels is usually not high. Reversible adhesion can also be achieved by adjusting cohesion force of the gels,^[^ ^5^ ^]^ since adhesion is determined by not only adhesion force between the gel and the substrate but also the cohesion force of the gel.^[^ ^14^, ^15^ ^]^ When peeling a gel off the substrate, the gel around the interface will be deformed. A tough gel will be able to resist the deformation via the dissipation of a significant amount of mechanical energy. Therefore a high toughness of the gel will contribute significantly to its adhesion strength.^[^ ^14^ ^]^ Simulation of the peeling process using finite‐element model further revealed that the adhesion strength increases monotonically with interfacial adhesion force, and the effect of interfacial adhesion force is dramatically augmented when the cohesion force of the gel is enhanced.^[^ ^14^ ^]^ Previously the mechanical properties of the gels were tuned using liquid‐to‐solid phase transition of the polymers,^[^ ^4^, ^11^ ^]^ drying and wetting of poly(2‐hydroxyethyl methacrylate) hydrogel,^[^ ^16^ ^]^ and reversible forming and breaking of crosslinks.^[^ ^17^ ^]^ In some cases, strong adhesion at attachment state was achieved.^[^ ^16^, ^17^ ^]^ Unfortunately these gels usually suffer from the residue left on the contact surface after detachment, because their failure mode is usually cohesive.^[^ ^4^ ^]^ Up to now, it still remains a great challenge to achieve strong and reversible hydrogel adhesion.^[^ ^3^, ^16^ ^]^ In addition, hydrogel adhesives usually suffer from poor environmental tolerances because of their dehydration in open air.^[^ ^18^ ^]^

To overcome the two challenges here a new reversible gel adhesive was designed using a new switching mechanism and new solvent. Deep eutectic solvents (DESs) are new generation green solvents with merits including nontoxicity, low cost, and easy preparation.^[^ ^19^, ^20^ ^]^ Taking advantage of their low vapor pressure, they were used to prepare eutectogels with excellent anti‐drying properties.^[^ ^21^, ^22^ ^]^ However most DES are hydrophilic and absorb moisture from the air. Here a hydrophobic DES was used to further improve the environmental stability of the resulting eutectogels.^[^ ^23^, ^24^ ^]^ We reported the first example of UCST (upper critical solution temperature)‐type thermosensitive behaviors of a polymer in DES. Unlike previous approaches here we used the thermally induced solvophilicity‐solvophobicity phase transition to adjust the mechanical properties of the eutectogels. Accordingly the gels can be reversibly switched between attachment and detachment states. The adhesive properties of the gel were further improved by the introduction of a novel mechanism of energy dissipation, i.e., energy dissipation through the breakage of intramolecular hydrogen bonds in the α‐helical peptide chains.^[^ ^21^, ^25^ ^]^ Finally a switchable gel adhesive which can be facilely switched by temperature, with high switching ratio, high maximum adhesion and high environmental stability, and allowing for repeated attaching/detaching, was obtained. Soft gripper with high load‐bearing capability and climbing robot capable of climbing on vertical and inverted surfaces were successfully designed using the new switchable gel adhesive.

## 2 Results and Discussion

### 2.1 Thermosensitive Behaviors of Poly(Benzyl Acrylate) in Menthol (Men):Thymol (Thy) DESs

**Figure 1:**
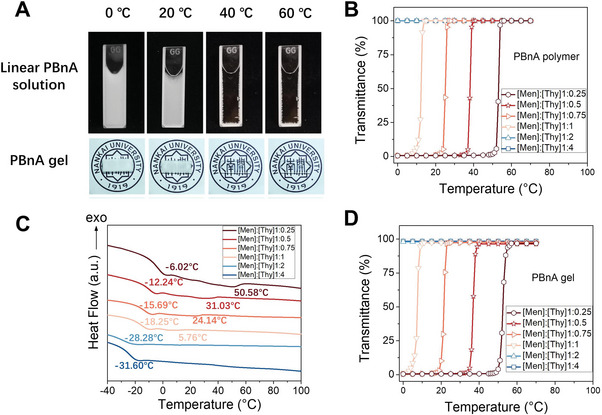
A) Photographs of linear PBnA solution and gel at various temperatures. The solvent is menthol:thymol DES with a [Men]:[Thy] molar ratio of 1:0.5. B) Light transmittance of PBnA solutions in menthol:thymol DESs with various [Men]:[Thy] molar ratios as a function of temperature. λ = 500 nm. The concentration of PBnA was 4.0 g/mL. C) Differential scanning calorimetry (DSC) thermograms of PBnA solutions in menthol:thymol DESs with various molar ratios. D) Light transmittance of PBnA gel in menthol:thymol DESs with various molar ratios as a function of temperature. *λ* = 500 nm.

Numerous thermosensitive polymers capable of changing their solubility in response to temperature change have been reported in the literature.^[^ ^26^, ^27^ ^]^ They may exhibit either a lower critical solution temperature (LCST)^[^ ^28^, ^29^ ^]^ or an upper critical solution temperature (UCST). A well‐known LCST‐type thermosensitive polymer is poly(N‐isopropylacrylamide), which dissolves well in water at room temperature, but precipitates out from the solution when heated above its LCST (≈32 °C).^[^ ^30^, ^31^ ^]^ In contrast, UCST‐type polymers dissolve well at high temperatures but phase separation occurs when cooled blow their UCST.^[^ ^26^ ^]^ These thermosensitive behaviors were usually observed when they dissolve in water or conventional organic solvents. Interestingly here UCST‐type thermosensitive behaviors were observed from poly(benzyl acrylate) (PBnA) solution in menthol:thymol DESs. As a typical example, the PBnA solution in DES with a [Men]: [Thy] molar ratio of 1:0.5 is clear at 60 and 40 °C but turns white when cooled to 20 and 0 °C (**Figure** **1**A). The results indicate the polymer has a high solubility in the solvent at high temperatures, but a low one at low temperatures. Therefore the polymer precipitates out from the solution when cooled. The thermosensitive behavior was further studied by turbidity. As shown in Figure 1B, upon cooling the transmittance of the solution drops sharply from ∼100% to close to 0. The UCST of the polymer in this solvent was determined to be 38 °C. The transition is not only quite sharp but also reversible (Figure S1, Supporting Information). To the best of our knowledge, this is the first time to observe UCST‐type thermosensitive behaviors from a polymer solution in DESs. Previously the same DES was used as solvent for atom transfer radical polymerization of various monomers, including methyl acrylate, methyl methacrylate, 2‐(dimethylamino) ethyl methacrylate, glycidyl methacrylate, 2‐hydroxyethyl acrylate, hydroxyethyl methacrylate and poly(ethylene glycol) methyl ether acrylate. No phase separation was observed in these systems.^[^ ^32^ ^]^ We also tested if phase transition occurs in poly(butyl acrylate) solution in menthol:thymol DESs. Again no phase separation was observed in the temperature range from 0 to 70 °C (Figure S2, Supporting Information). PBnA solutions in other DESs, including menthol: decanoic acid, tetraoctylammonium chloride: glycerol, and tetrabutylammonium chloride: lauric acid, do not present thermosensitive behaviors either.

The thermosensitive behavior of PBnA in DESs with other [Men]: [Thy] molar ratios was also studied. Similar reversible UCST behaviors were observed in DESs with a [Men]: [Thy] molar ratio of 1:0.25, 1:0.75 and 1:1, but not observed in DESs with a [Men]: [Thy] molar ratio of 1:2 and 1:4 (Figures S3 and S1, Supporting Information; Figure 1B). The UCST of PBnA decreases with increasing thymol content in the solvent (Figure S4, Supporting Information). The result may be explained by the similarity‐intermiscibility theory. Both PBnA and thymol have benzyl groups. Because of their structural similarity, increasing thymol content in the solvent leads to an increasing solubility of PBnA, and hence deceasing UCST of the polymer. The thermal behaviors were also studied by differential scanning calorimeter (DSC) (Figure 1C). For the PBnA solution in DESs with a [Men]:[Thy] molar ratio of 1:0.25, 1:0.5, 1:0.75 and 1:1, an endothermic peak was observed, confirming again the occurrence of phase transition in these solvents. The UCSTs determined by DSC is close to the ones determined by turbidity (Figure S4, Supporting Information). Again, the UCST of the polymer decreases with increasing thymol content in the solvent. A series of PBnAs with different Mws were synthesized by RAFT polymerization according to ref. [33] (Table S1, Supporting Information). The UCST does not vary with the Mw of PBnA (Figure S5, Supporting Information).

The thermosensitive behavior of PBnA in menthol:thymol DESs makes it possible to synthesize thermosensitive eutectogels. Simply the monomer benzyl acrylate (BnA), the cross‐linker ethylene glycol dimethacrylate (EGDMA), and the photo‐initiator 1173 were dissolved in DESs and then UV‐cured. As expected, the resulting eutectogels exhibit similar thermosensitive behaviors with the linear polymer. The gels using DESs with [Men]:[Thy] molar ratios of 1:0.25, 1:0.5, 1:0.75, and 1:1 exhibit UCST‐type phase transition, while the ones using DESs with [Men]:[Thy] molar ratios of 1:2 and 1:4 do not (Figure 1A,D; Figure S6, Supporting Information). The UCSTs of the gels are close to that of the polymer and also decrease with increasing thymol content in the solvent (Figure S4, Supporting Information).

### 2.2 Switchable Adhesion of EGDMA‐Crosslinked PBnA Eutectogels

**Figure 2:**
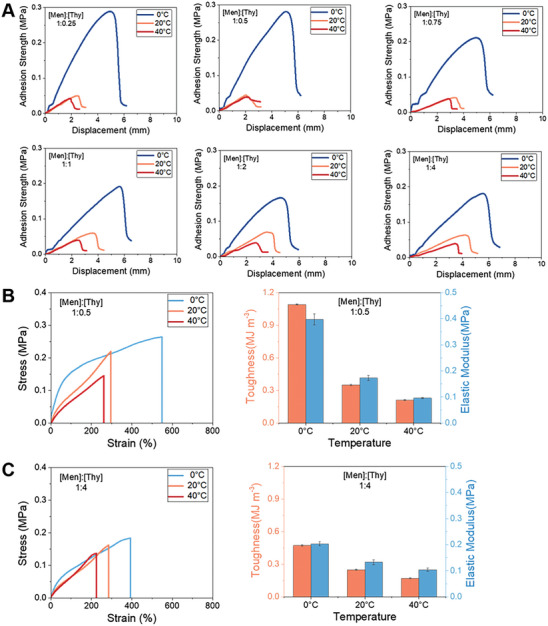
A) Shear peel curves of PBnA eutectogel prepared in DES with various [Men]:[Thy] molar ratios at 0, 20, and 40 °C. B) Tensile stress‐strain curves (left) and mechanical properties (right) of PBnA eutectogel prepared in DES with a [Men]:[Thy] molar ratio of 1:0.5. C) Tensile stress‐strain curves (left) and mechanical properties (right) of PBnA eutectogel prepared in DES with a [Men]:[Thy] molar ratio of 1:4.

The thermosensitive behavior makes the PBnA eutectogels potential switchable adhesive materials. For this purpose, their adhesive properties were investigated by lap shear tests using glass slides as adherends at different temperatures. As shown in **Figure** **2**A, all the gels present a higher adhesion strength at 0 °C but much lower adhesion strength at 40 °C. Switching ratio, defined as the ratio of adhesion strength at 0 °C and adhesion strength at 40 °C, was plotted in Figure S7 (Supporting Information). The gels exhibiting thermosensitive behaviors exhibit a relatively high switching ratio, while the gels that do not present the UCST behavior present a relatively low switching ratio. Among them, the eutectic gel with a [Men]:[Thy] molar ratio of 1:0.5 presents the highest switching ratio of 7.3.

The thermal switchable adhesion strength of the PBnA eutectogels should be mainly attributed to their thermal switchable mechanical properties. The mechanical properties of the gels were measured by uniaxial tensile tests. As shown in Figure 2B, for the gel in DES with a [Men]:[Thy] molar ratio of 1:0.5, when lowering temperature from 40 to 0 °C, elastic modulus increases by 3.16 times, and toughness increases by 4.23 times. When the gel is cooled from 40 to 0 °C, the solvophilic PBnA polymer chains become solvophobic, leading to the association among the polymer chains. These chain aggregates act as additional physical cross‐links and thus stiffen the gel. The increased modulus may not be attributed to solvent extrusion as this phenomenon was not observed during the cooling process. In contrast for the gel with a [Men]:[Thy] molar ratio of 1:4, because the polymer is non‐thermosensitive in the solvent, a much smaller change in modulus and toughness was observed when temperature is lowered from 40 to 0 °C (Figure 2C). Similar to tensile tests, compression tests, dynamic mechanical analysis tests and rheological measurement also revealed a more significant increase in compressive strength and storage modulus of the gel in DES with a [Men]:[Thy] molar ratio of 1:0.5 than the one a [Men]:[Thy] molar ratio of 1:4 when cooling from 40 to 0 °C (Figures S8, S9, and S10, Supporting Information). Besides these two gels, other gels also follow the similar trend, i.e., the thermosensitive gels exhibit a more significant change in mechanical properties than the non‐thermosensitive ones (Figure S11, Supporting Information).

To further verify that the thermal switchable adhesion of PBnA eutectogels originates from the thermosensitive behavior of PBnA in menthol:thymol DES, EGDMA‐crosslinked poly(butyl acrylate) eutectogels were also synthesized. Similar to the linear poly(butyl acrylate) polymer, no UCST behavior was observed from poly(butyl acrylate) eutectogels (Figure S12, Supporting Information). As a result, lowering temperature from 40 to 0 °C does not significantly increases the adhesion strength of the gels (Figure S13, Supporting Information). The switching ratio is only in the range of 1.5–2 (Figure S14, Supporting Information).

Since the PBnA gel in DES with a [Men]:[Thy] molar ratio of 1:0.5 exhibits the best switchable adhesion, this solvent was used for further studies. To study the effect of crosslink densities, a series of PBnA eutectogels with various crosslink densities were synthesized. Switchable adhesion was observed from all the gels (Figure S15, Supporting Information). The switching ratio first increases with increasing EGDMA content and then decreases with increasing EGDMA content (Figure S16, Supporting Information). Increasing crosslinking density increases the stiffness of the gel, and therefore increases its ability to resist external forces during peeling. However, increasing crosslinking density also restricts the movement of the polymer chains. The two opposite trends lead to a maximum switching ratio at a crosslinker content of 0.75%. Switchable adhesion was also observed from PBnA eutectogels with different DES contents (Figure S17, Supporting Information). When a small amount of DES was used (0.25 and 0.5 g), the resulting gels exhibit a high switching ratio (Figure S18, Supporting Information). However, increasing the amount of DES to 1.0 g significantly reduces the switching ratio, because of the reduced solid content of the gel and hence reduced cohesion. Among all the eutectogels, the B_2_E_0.75%_H_0.5_ gel with a crosslinker content of 0.75% and a DES content of 0.5 g was found to present the best switchable adhesion properties.

### 2.3 Improved Properties of Peptide‐Crosslinked Poly(Benzyl Acrylate) Eutectogels

Our objective is not only to achieve switchable adhesion, but also high adhesion strength at attachment state. When cooled to 0 °C a high adhesion strength of 0.28 MPa was observed for B_2_E_0.75%_H_0.5_ gel. In order to further increase the adhesion strength at attachment state, we tried to introduce mechanics of energy dissipation. Previous studies reveal that energy dissipation delays the debonding of a gel from an adherend when separating them.^[3,^ ^34^, ^35^ ^]^ Various mechanics of energy dissipation were used to achieve strong adhesion.^[3,^ ^34^ ^]^ Here to introduce mechanics of energy dissipation, α‐helical peptide chains were introduced into the gel by replacing the crosslinker EGDMA with a poly(benzyl L‐glutamate)‐based peptide crosslinker (PC).^[^ ^25^ ^]^ The synthesis and characterization of the PC was collected in Figure S19 and Table S2 (Supporting Information). The weight‐average molecular weight of the PC was measured to be 4.8×10^3^ by GPC. The degree of polymerization was measured to be 22 (Table S2, Supporting Information). CD study reveals a α‐helical structure of the crosslinker in menthol:thymol DES (Figure S20A, Supporting Information).

**Figure 3:**
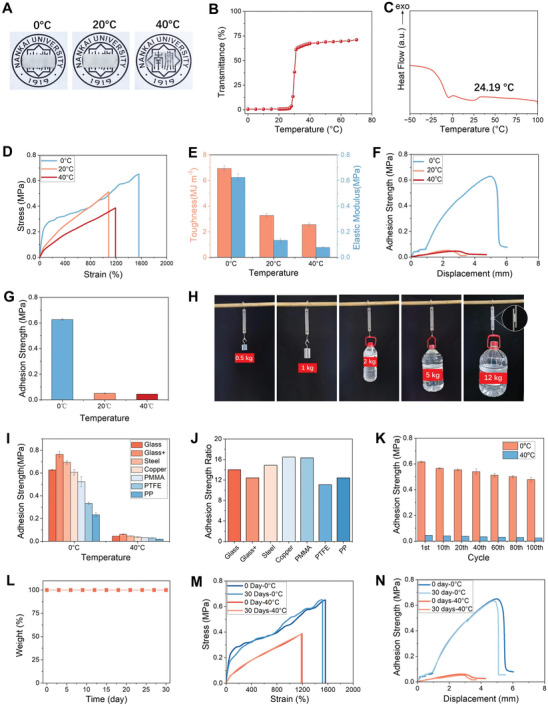
A) Photographs of a B_2_PC_0.75%_H_0.5_ gel taking at various temperatures. B) Light transmittance of a B_2_PC_0.75%_H_0.5_ gel as a function of temperature. *λ* = 500 nm. C) DSC thermograms of a B_2_PC_0.75%_H_0.5_ gel. D,E) Tensile stress–strain curves (D) and mechanical properties (E) of a B_2_PC_0.75%_H_0.5_ gel measured at various temperatures. F,G) Shear peel curves (F) and adhesion strength (G) of a B_2_PC_0.75%_H_0.5_ gel measured at various temperatures. H) Lifting cargos with different weights using a B_2_PC_0.75%_H_0.5_ gel. The gel size was 25 mm ×10 mm× 1 mm. I,J) Adhesion strength (I) and switching ratio (J) of B_2_PC_0.75%_H_0.5_ gel on various substrates. K) Adhesion strengths of a B_2_PC_0.75%_H_0.5_ gel at 0 °C and 40 °C repeatedly measured for 100 times. L) Weight change of a B_2_PC_0.75%_H_0.5_ gel stored under ambient conditions. M,N) Tensile stress–strain curves (M) and shear peel curves (N) of a B_2_PC_0.75%_H_0.5_ gel before and after 30 days storage under ambient conditions.

Similar to EGDMA‐crosslinked eutectogel, the peptide cross‐linked eutectogels also exhibit UCST behavior. It is transparent at 40 °C but turns white when cooled to 20 and 0 °C (**Figure** **3**A). The UCST was measured to be 30 °C by turbidity and 24.2 °C by DSC (Figure 3B,C). As expected, replacing EDGMA with PC significantly improves the mechanical properties of the gel (Figure 3D,E). At 0 °C the elastic modulus increases from 0.398 MPa for EGDMA‐crosslinked gel to 0.626 MPa for peptide‐crosslinked gel, and toughness increases from 1.10 MJ m^−3^ for EGDMA‐crosslinked gel to 6.95 MJ m^−3^ for peptide‐crosslinked gel. Similar results were previously observed in peptide‐crosslinked hydrogels and eutectogel.^[^ ^21^, ^25^, ^36^ ^]^ As previous studies revealed, the introduction of α‐helical peptide chains into the gel network provides a novel mechanism for energy dissipation, i.e., energy dissipation via the breakage of intramolecular hydrogen bonds stabilizing the α‐helix,^[^ ^25^ ^]^ and thus toughens the gels. As shown in Figure S20B (Supporting Information) stretching the gel breaks the intramolecular hydrogen bonds and hence the α‐helical structure, which was confirmed by the dramatic change in the CD spectra. The CD spectra recovered immediately upon unloading, suggesting the reformation of the α‐helical structure. Because of the thermosensitivity of PBnA polymer in the gel, the peptide‐crosslinked gel also exhibits temperature‐tunable mechanical properties. As shown in Figure 3D,E, at 0 °C the toughness of the gel was 2.74 times higher than that at 40 °C, and the elastic modulus was ≈8 times higher than at 40 °C. As expected, the B_2_PC_0.75%_H_0.5_ gel also exhibits thermal switchable adhesion properties (Figure 3F,G). The adhesion strength reaches 0.627 MPa at 0 °C but only 0.045 MPa at 40 °C, leading to a switching ratio of 14.0. As a demonstration for its high adhesion strength at attachment state, two pieces of stainless steel were bonded together with a B_2_PC_0.75%_H_0.5_ gel and cooled to 0 °C. They were not debonded by a load up to 12 Kg (Figure 3H). However the load could be released successfully by heating the gel (Movie S1, Supporting Information). The significantly improved adhesion strength of the peptide‐crosslinked gel at 0 °C should be attributed to its improved mechanical strength.

The adhesion strength of B_2_PC_0.75%_H_0.5_ gel at attachment state is not only higher than the B_2_E_0.75%_H_0.5_ gel, but also much higher than most switchable adhesive gels reported in the literature^[^ ^5^, ^6^, ^8^, ^9^, ^10^, ^11^, ^37^, ^38^, ^39^, ^40^, ^41^, ^42^ ^]^ (Table S3, Supporting Information). For example, using dynamic multiscale contact synergy Zhou et al^[^ ^5^ ^]^ designed a large‐span switchable adhesive hydrogel. The adhesion strength at attachment state of the gel was reported to be only ≈21 kPa. In another example, Dai et al^[^ ^8^ ^]^ designed a hydrogel that can switch between adhesive and nonadhesive states rapidly in response to electrical stimulus. However, the maximum adhesion strength is only 11.5 kPa.

Besides glass, the gel also exhibits switchable adhesion on many other substrates, including positively charged glass, metals, and even polymeric materials with low surface energy, such as PMMA and PTFE (Figure 3I). This property allows the gel to be attached and unattached on these substrates on demand by changing temperature. The gel exhibits the highest adhesion strength at attachment state on positively charged glass, but the highest switching ratio was observed on copper (Figure 3J).

More importantly, the gel can be attached and detached repeatedly. As shown in Figure 3K, the adhesion strengths of the gel at 0 and 40 °C were repeatedly measured by lap shear tests. After 100 cycles the adhesion strength of the gel at attachment state still reaches 0.47 MPa. The excellent reusability of the gel was first attributed to that its failure mode is adhesive, not cohesive. After detachment, the gel remains intact and no residue is left on the substrate (Figure S21, Supporting Information). Very differently, the previously reported reversible adhesive gels based on tunable cohesion force usually have a cohesive failure mode, and therefore suffer from the residue left on the contact surface after detachment.^[^ ^4^ ^]^ Unlike other mechanisms for energy dissipation, which either are irreversible or only partially reversible and lead to significantly reduced mechanical strength after unloading, the mechanism for energy dissipation used here is fully reversible.^[^ ^25^ ^]^ Upon unloading, the fractured intramolecular hydrogen bonds will fully reform to allow the precise refolding back of the α‐helical structure (Figure S20B, Supporting Information).^[^ ^25^ ^]^ The network of the gel and hence its mechanical strength will fully recover. Therefore, the adhesion strength of the gel will not decrease significantly in the following adhesion‐peeling cycles. The low vapor pressure of the solvent used here is also important for the excellent reusability of the gel because there is almost no solvent loss during the experiment period (≈12 h).^[^ ^20^, ^21^ ^]^

Because of the low vapor pressure of DES, the B_2_PC_0.75%_H_0.5_ gel also exhibits a long‐term stability. After the gel was stored for 30 days under ambient conditions, little change in its appearance was found (Figure S22, Supporting Information). The mass of the gel remained unchanged, (Figure 3L) suggesting no mass loss because of the low vapor pressure of the solvent. In contrast, dehydration usually occurs for hydrogels because of the evaporation of water during storage.^[^ ^21^ ^]^ The gel mass remained unchanged even after 30 days storage in a humid environment (Figure S23, Supporting Information). Most DES are hydrophilic and absorb moisture from the air.^[^ ^23^ ^]^ In contrast, the eutectic solvent used here is hydrophobic. Therefore, the gel was not affected by the small amount of water present in the environment. The gel remains to be thermosensitive after 30 days storage (Figure S22, Supporting Information). Little change in UCST was found (Figure S24, Supporting Information). Therefore the gel still presents thermally switchable mechanical and adhesive properties. As shown in Figure 3M, after 30 days storage the tensile strength, elongation at break, and toughness measured at both 0 and 40 °C remained almost unchanged. The adhesion strengths measured at both 0 and 40 °C are also almost equal to the corresponding values before storage (Figure 3N).

### 2.4 Applications in Soft Grippers and Climbing Robots

**Figure 4:**
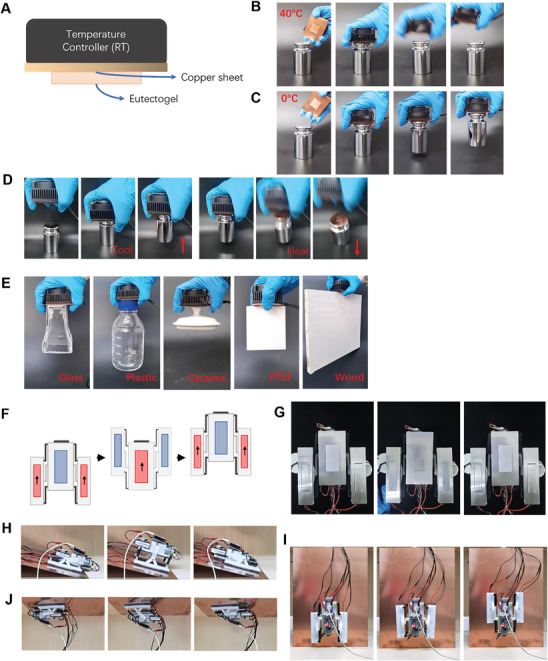
A) Schematic plot of the B_2_PC_0.75%_H_0.5_ gel‐based gripper. B,C) Lifting a 1 kg weight using the B_2_PC_0.75%_H_0.5_ gel‐based gripper at 40 °C (B) and 0 °C (C). D) Transporting a 1 kg weight using the B_2_PC_0.75%_H_0.5_ gel‐based gripper. E) Transporting various cargos using the B_2_PC_0.75%_H_0.5_ gel‐based gripper. F) Structure and movement principle of the climbing robots. G) Photographs of the climbing robot with B_2_PC_0.75%_H_0.5_ gels on the bottom of the feet. The gel temperatures were switched in harmony. H–J) Photographs of the robot climbing on an inclined (30°) (H), vertical (90°) (I), and an inverted copper surface (J).

The strong and switchable adhesion of B_2_PC_0.75%_H_0.5_ gel makes it highly potential for application as soft gripper in robots. Unlike the traditional rigid grippers, soft grippers are flexible and compliant, and have better adaptability to various target objects.^[^ ^43^ ^]^ To demonstrate the ability of B_2_PC_0.75%_H_0.5_ gel to capture, transport, and release a cargo, a piece of gel was fabricated on a piece of copper sheet (1 mm thick). The other side of the copper sheet was attached onto a temperature controller composed of a semiconductor and a heat sink. (**Figure** **4**A) Using this device, the gel temperature can be facilely switched between 0 and 40 °C. As shown in Figure 4B and Movie S2 (Supporting Information), when the gel is heated to 40 °C, it cannot lift the weight because of its low adhesion at this temperature. To lift the weight, the gel was first put on the weight to allow it to form conformal contact with the weight. Then the temperature was lowered to 0 °C and the weight was lifted successfully. (Figure 4C; Movie S3, Supporting Information) The weight was then transported to the desired place. To release the weight, the gel temperature was adjusted to 40 °C, and the cargo was detached by its own weight. The whole grasping, transporting, and releasing process was demonstrated in Figure 4D and Movie S4 (Supporting Information).

Besides metallic objects, other objects including plastics, woods, and ceramics can also be transported using the B_2_PC_0.75%_H_0.5_ gel‐based gripper. (Figure 4E) Because of the high adhesive strength of the gel at attachment state, the gripper can be used to transport objects with a high mass. In the above example, the mass of the weight is 1 kg. The maximum mass transported using the gripper is 4 kg (Figure S25, Supporting Information). The load‐bearing capacity of the gripper designed here is much higher than other adhesive gel‐based soft grippers reported previously. For example, Eklund et al designed a hydrogel gripper which can only transport lightweight cargos with a mass up to 2.1 g.^[^ ^10^ ^]^ In another report, the gripper can be used to transport a metal block of ≈200 g.^[^ ^6^ ^]^

The switchable adhesion of B_2_PC_0.75%_H_0.5_ gel also makes it possible to design climbing robots moving on wall and ceiling in a manner similar to gecko.^[^ ^1^ ^]^ As shown in Figure 4F, a climbing robot was designed using a mechanism and movement principle similar to the one previously designed by Huang et al.^[^ ^8^ ^]^ Briefly, the robot equips with one central foot and two side feet. The robot is driven to move forward by alternate lifting and putting down the central foot and the side feet. On the bottom of each feet, a temperature controller and a piece of B_2_PC_0.75%_H_0.5_ gel were assembled. Before lifting a foot, the gel temperature is raised to ≈40 °C by the temperature controller to lower the adhesion force between the foot and the substrate to facilitate the lifting of the foot. When the foot is put down, the gel temperature is lowered to ≈0 °C by the temperature controller to dramatically increase the adhesion force between the foot and the substrate, and the foot will adhere to the substrate firmly. The movement and temperature change of the central foot and side feet were harmonized. So when lifting the central foot, the gels on the side feet are cooled to ≈0 °C to adhere the side feet firmly on the substrate, and vice versa (Figure 4G). In this way, the central foot and the side feet can be lifted, protruded forward, and put down alternately, thus driving the robot to move forward. The robot can not only climb on horizontal and sloping surface, but also on vertical and inverted surfaces (Figure 4H–J; Movies S5 and S6, Supporting Information.). The average velocity of movement was ≈6 cm min^−1^ when crawling vertically, while the velocity was ≈4.5 cm min^−1^ when crawling on inverted surface. It is noteworthy that the strong adhesion of B_2_PC_0.75%_H_0.5_ gel on attachment state allows it to drive the movement of a heavy robot. The weight of the robot, including the temperature control system and gels, is ≈550 g, while the weight of robot designed by Huang et al is only ≈113 g.^[^ ^8^ ^]^

## 3 Conclusion

In summary, for the first time, we observed UCST‐type phase transition of PBnA polymer and gel in menthol:thymol DESs. The polymer dissolves well in the DES at high temperature but becomes solvophobic when cooled. The UCST of the polymer decreases with increasing thymol content in the solvent. The temperature‐induced solvophilicity to solvophobicity transition leads to significant change in the mechanical properties of PBnA eutectogels, which further induces significant change in their adhesion properties. The switchable mechanical and adhesion properties of the gels were further improved by replacing the crosslinker EGDMA with a peptide crosslinker, which allows energy dissipation via the breakage of intramolecular hydrogen bonds stabilizing the α‐helical structure of the peptide chains. The resulting peptide‐crosslinked PBnA eutectogel can be easily switched between attachment and detachment states by temperature, with a high switching ratio of 14.0. In addition, its adhesion strength at attachment state reaches 0.627 MPa, which is much higher than most of the reversible adhesive hydrogels. Reversible adhesion of the eutectogel was observed on various substrates. More importantly because of its adhesive failure mode, the reversible energy dissipation, and low vapor pressure of DES, the eutectogel can be repeatedly switched between attachment and detachment states. The low vapor pressure of DES also endows excellent long‐term stability of the gel. The gel was used as soft gripper to lift, transport, and release heavy cargos. In addition, a climbing robot capable of moving on vertical and inverted surface in a manner similar to gecko was designed.

## 4 Experimental Section

### Materials

Benzyl acrylate (BnA) and DL‐menthol (99%) were purchased from Adamas. Ethylene glycol dimethacrylate (EGDMA, 99%) and thymol (>99.0%) were purchased from Aladdin. γ‐Benzyl‐L‐glutamate N‐carboxyanhydride (BLG‐NCA, 97%) were purchased from Bidepharm. 1‐Hydroxybenzotriazole (HOBT, 99%), 2‐hydroxy‐2‐methylpropiophenone (photoinitiator 1173, 97%) were purchased from Crgent Biotech. N‐ethyl‐N'‐(3‐dimethylaminopropyl)carbodiimide hydrochloride (EDCI, 99%) and N, N‐dimethyl formamide (DMF, 99.9%, dry) were purchased from Tianjin Heowns Biochem LLC. 3‐Buten‐1‐amine (99%) was purchased from Sigma‐Aldrich. Acrylic acid (98%) was purchased from Tianjin Chemical Reagents Co. 2,2′‐Azoisobutyronitrile (AIBN) was purchased from TCI.

### Preparation of Menthol:Thymol DESs

The menthol:thymol DESs was prepared by mixing DL‐menthol and thymol and stirring at 50 °C until a clear liquid was obtained. By using different molar ratio of menthol and thymol, menthol:thymol DESs with different [Men]:[Thy] molar ratios were prepared.

### Synthesis of Linear Poly(Benzyl Acrylate) (PBnA) Polymer

To prepare linear PBnA polymer, BnA monomer (5.0 g, 30.75 mmol) was dissolved in dry ethyl acetate (40 ml) and deoxygenated by nitrogen bubbling. AIBN (12.62 mg, 0.077 mmol) was then added and the polymerization was carried out at 60 °C for 12 h in N_2_ atmosphere. The polymerization was stopped by cooling the solution in ice water and exposing to air. The resulting product was precipitated in excess of cold ethyl ether and dried overnight under reduced pressure at room temperature. Yield: 68.45%. The molecular weight of the product was determined by GPC to be 6.90×10^4^.

### Synthesis of EGDMA‐Crosslinked PBnA Eutectogels

To synthesize the EGDMA‐crosslinked PBnA eutectogels, the monomer BnA, the crosslinker EGDMA, and photo‐initiator 1173 (5.0 mg) were dissolved in DES. The solutions were then purged with nitrogen and cured by 5 min UV irradiation. The resulting gels were named as B_x_E_y_H_z_, where B represents the monomer BnA, x represents the mass of monomer, E represents the cross‐linker EGDMA, y represents the molar ratio of cross‐linker to monomer, H represents the hydrophobic DESs, and z represents the mass of DES. For example, the B_2_E_0.75%_H_0.5_ gel was synthesized by polymerization of a pregel solution containing 2.0 g of BnA and 0.5 g of DES, and the molar ratio of EGDMA to BnA is 0.75%.

### Synthesis of Peptide‐Crosslinked PBnA Eutectogels

The peptide crosslinker (PC) was synthesized by ring‐opening polymerization of BLG‐NCA according to ref. [44]. Briefly, BLG‐NCA (5.26 g, 20.0 mmol) was dissolved in dried DMF (80 mL) at room temperature under N_2_ atmosphere. The polymerization was initiated by the addition of 3‐buten‐1‐amine (78 µL, 0.8 mmol). The reaction was allowed to proceed at room temperature for 72 h. Then acrylic acid (547 µL, 8.0 mmol), EDCI (379 mg, 2.0 mmol), and HOBT (105 mg, 0.8 mmol) were added into the reaction mixture to cap the amino end. After stirring for additional 48 h, the product was precipitated in excess amount of diethyl ether and dried overnight under vacuum. Yield: 75.89%. The molecular weight of the product was determined by GPC to be 4.9×10^3^ and the degree of polymerization (DP) was determined to be 22.

To synthesize the peptide‐crosslinked eutectogels, BnA (2.0 g, 12.3 mmol), crosslinker PC (0.375 g, 0.92 mmol), and photo‐initiator 1173 (5.0 mg) were dissolved in DES (0.50 g). The solutions were purged with nitrogen and then cured by 5 min UV irradiation. The resulting gels were named as B_2_PC_0.75_H_0.5_, in the same way as the EGDMA‐crosslinked gels.

### Mechanical Tests

Mechanical tests were performed on a UTM6103 testing machine (Shenzhen Suns Technology Stock Co., Ltd, China). If not otherwise specified, all tensile tests were performed at room temperature and a speed of 50 mm min^−1^. The samples for tensile tests were made in a dumbbell shape of type III in accordance with GB/T528‐2009 standard, which is equivalent to ISO 37:2005. All tests were repeated at least four times.

The adhesion strength of the gels was measured by lap shear tests (ASTM F2255) using the same machine at a speed of 10 mm min^−1^. All gel samples had a size of 25 mm × 10 mm × 1 mm. Commercial glass slides were used as adherends. Each test was repeated five times.

### Other Characterizations

^1^H NMR spectra was measured on a Bruker AV400 spectrometer. GPC measurements were performed on a Hitachi L‐2490 using DMF as the eluent (flow rate = 1 mL min^−1^, T = 40 °C) and polymethylmethacrylate (PMMA) as standard. Transmittance of the samples was measured on a Shimadzu TCC‐240A UV–vis spectrophotometer at 500 nm. Circular dichroism (CD) spectra was obtained using a BioLogic MOS‐500 circular dichroism spectrometer with a 150 W Xenon lamp. DSC were measured on a Netzsch DSC3500 Sirius thermal analysis system using a heating rate of 10 °C min^−1^.

## Conflict of Interest

The authors declare no conflict of interest.

## Supporting information

Supporting Information

Supplemental Movie 1

Supplemental Movie 2

Supplemental Movie 3

Supplemental Movie 4

Supplemental Movie 5

Supplemental Movie 6

## Data Availability

The data that support the findings of this study are available from the corresponding author upon reasonable request.
