# Supplementary material for: Strong and Thermo‐Switchable Gel Adhesion Based on UCST‐Type Phase Transition in Deep Eutectic Solvent
Source: Adv Sci (Weinh). 2024 Jun 17;11(31):2400938. doi: 10.1002/advs.202400938 (PMC11336952; doi:10.1002/advs.202400938)
Supplement: Supplementary file 1 — Supporting Information [file ADVS-11-2400938-s004.docx]

Supporting Information

Strong and thermo-switchable gel adhesion based on UCST-type phase transition in deep eutectic solvent

Huiyao Xu, Haocheng Li, Yan Zhang, Ying Guan,* Yongjun Zhang*


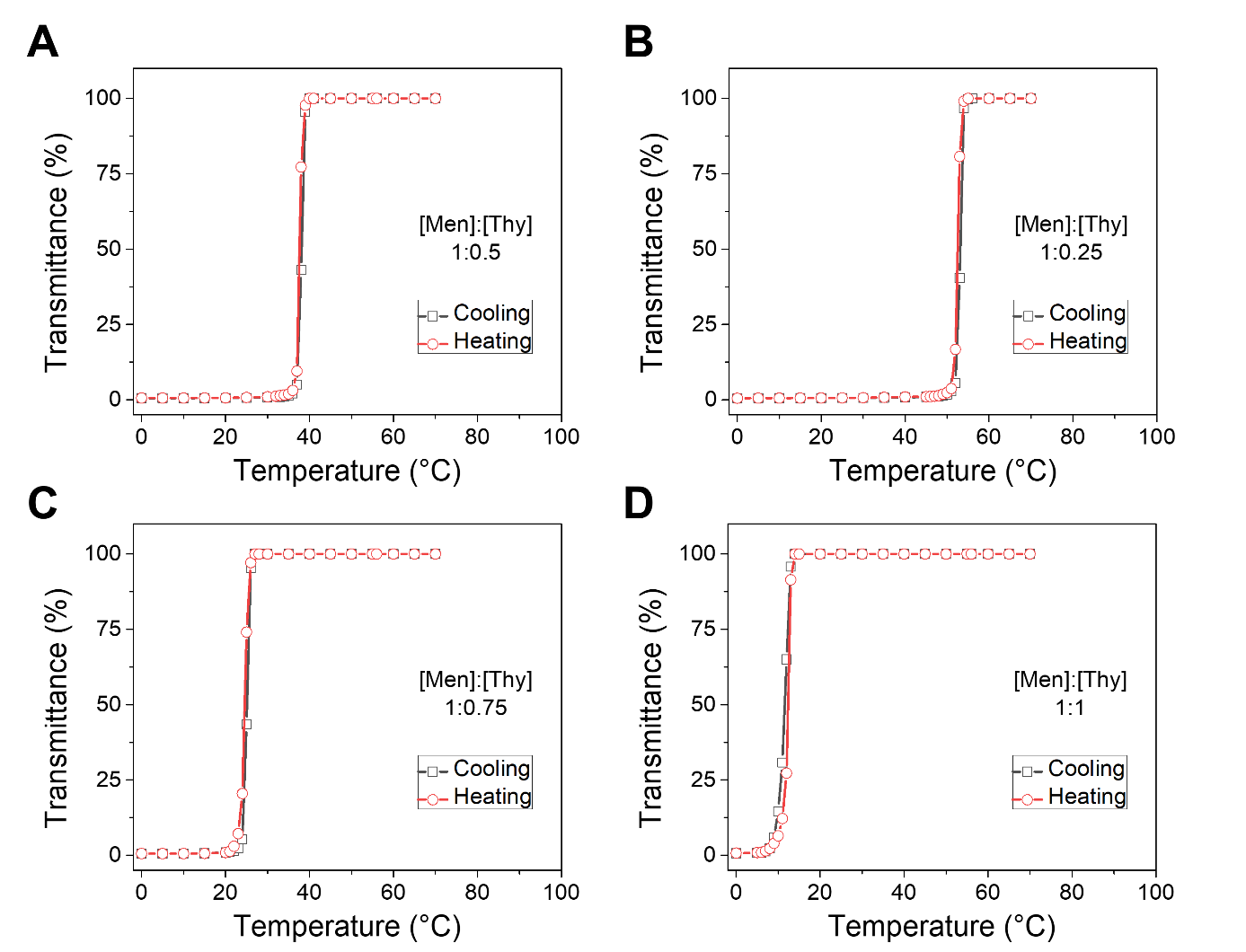


**Figure S1**. Light transmittance of PBnA solution in menthol:thymol DESs with a [Men]:[Thy] molar ratio of 1:0.5 (A), 1:0.25 (B), 1:0.75 (C) and 1:1 (D). λ = 500 nm. The concentration of PBnA was 4.0 g/mL.


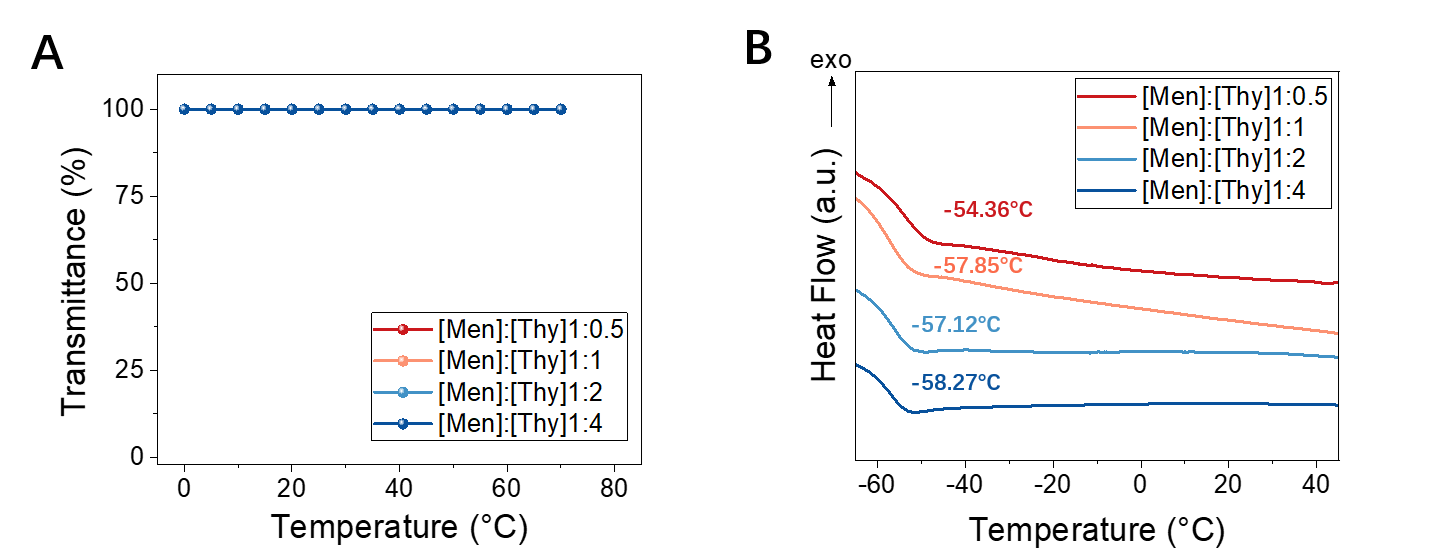


**Figure S2**. (A) Light transmittance of poly(butyl acrylate) solution in menthol:thymol DESs at various temperatures. λ = 500 nm. The polymer concentration was 4.0 g/mL. (B) DSC thermograms of poly(butyl acrylate) solutions in menthol:thymol DESs. Unlike PBnA, this polymer does not present thermosensitive behaviors in menthol:thymol DESs.


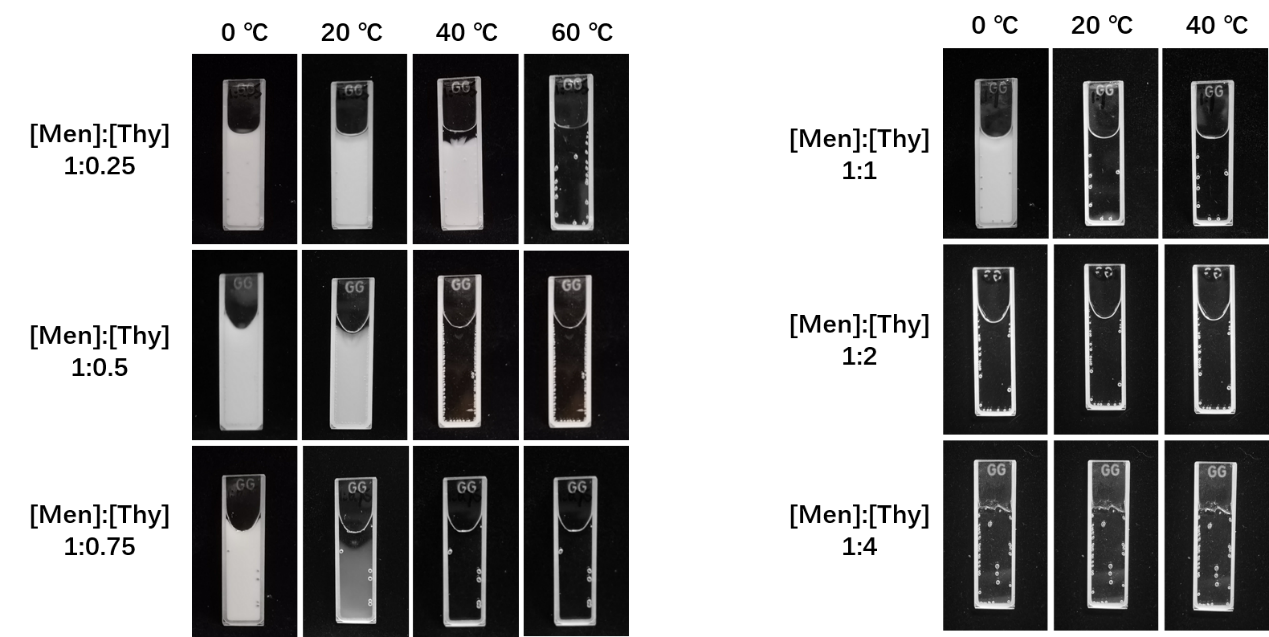


**Figure S3**. Photographs of linear PBnA solution in menthol:thymol DESs at different temperatures. [Men]:[Thy] molar ratio of the solvents was labelled.

**Figure S4**. UCSTs of linear PBnA or PBnA gel in menthol:thymol DESs with different [Men]:[Thy] molar ratios determined by turbidity or DSC.

**Figure S5**. Light transmittance of PBnA solutions in menthol:thymol DES with a [Men]:[Thy] molar ratio of 1:0.5. The degree of polymerisation (DP)s of PBnA were 50, 100, 150 and 200. λ = 500 nm. The concentration of PBnA was 4.0 g/mL.


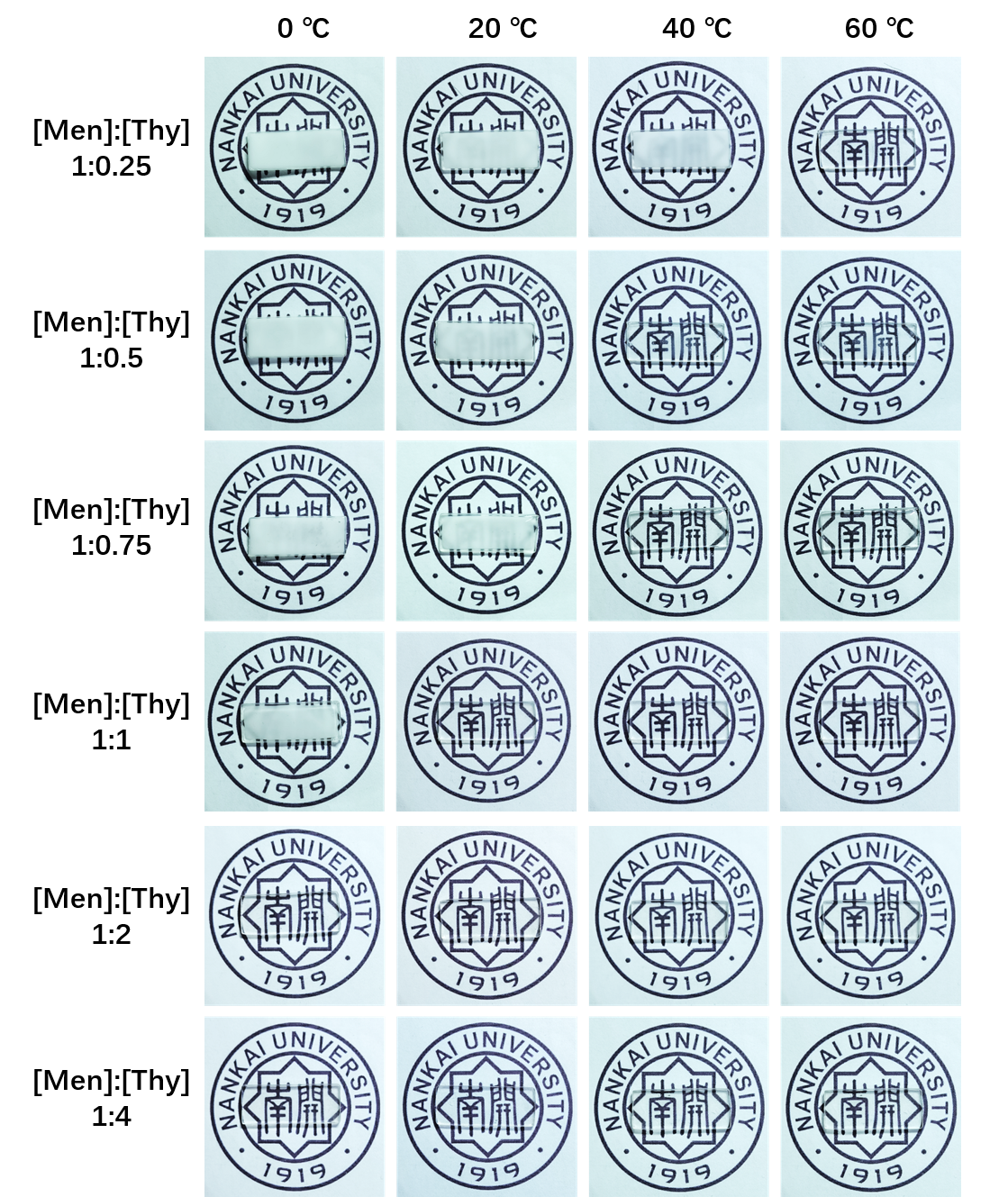


**Figure S6**. Photographs of PBnA gels at various temperatures. The gels were prepared in menthol:thymol DESs with various [Men]:[Thy] molar ratios as labelled. The crosslinker was EGDMA.

**Figure S7**. Switching ratio (the ratio of adhesion strength at 0°C and adhesion strength at 40°C) of EGDMA-crosslinked PBnA gel in menthol:thymol DESs with various [Men]:[Thy] molar ratios.


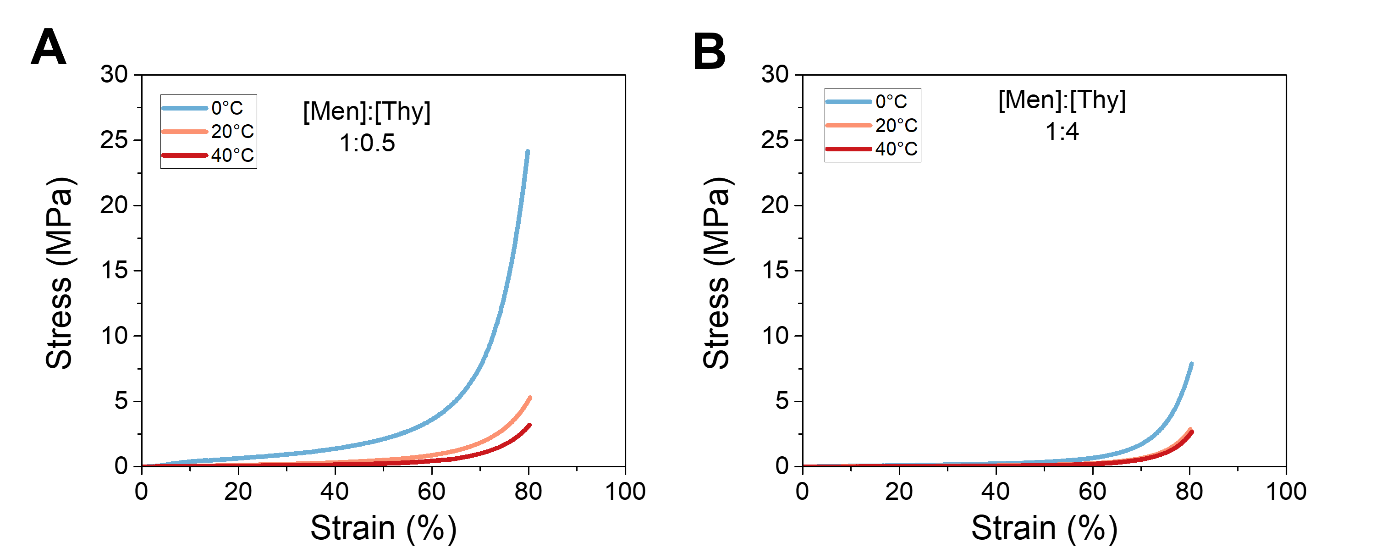


**Figure S8**. Compression curves of PBnA eutectogels prepared in DES with [Men]:[Thy] molar ratios of 1:0.5 (A) and 1:4 (B).


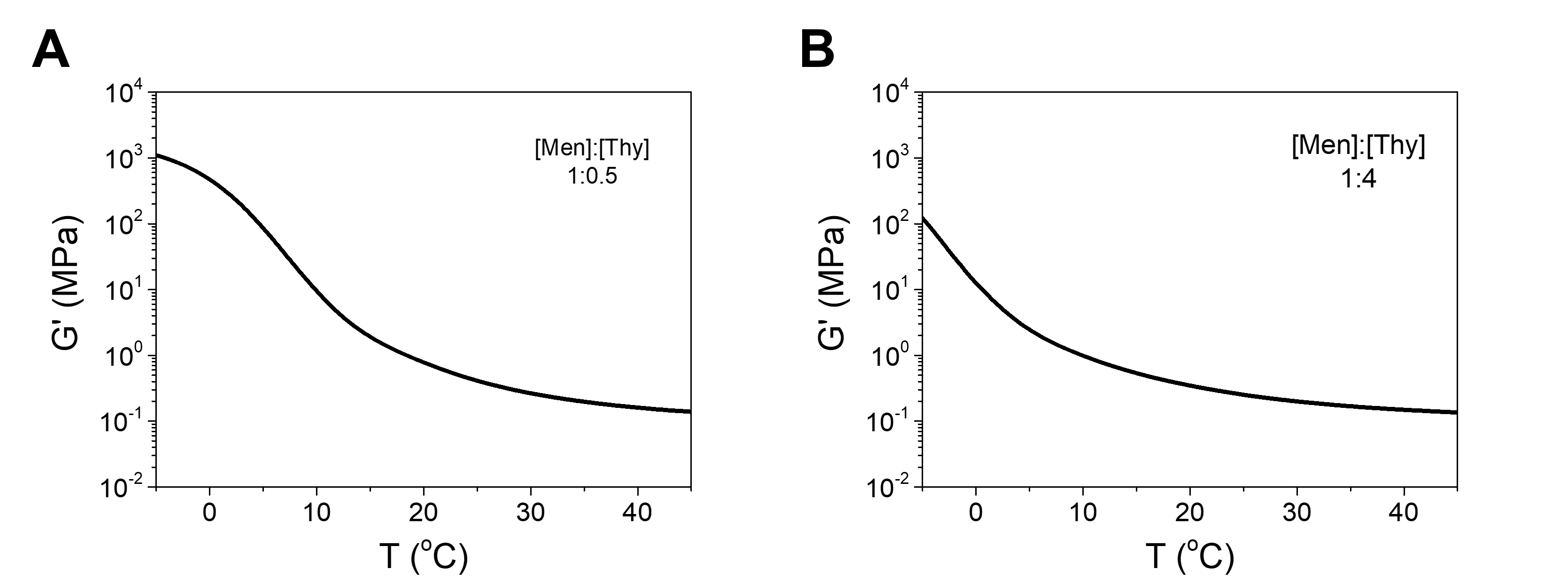


**Figure S9**. Storage moduli of PBnA eutectogels prepared in DES with [Men]:[Thy] molar ratios of 1:0.5 (A) and 1:4 (B) measured by dynamical mechanical analysis.


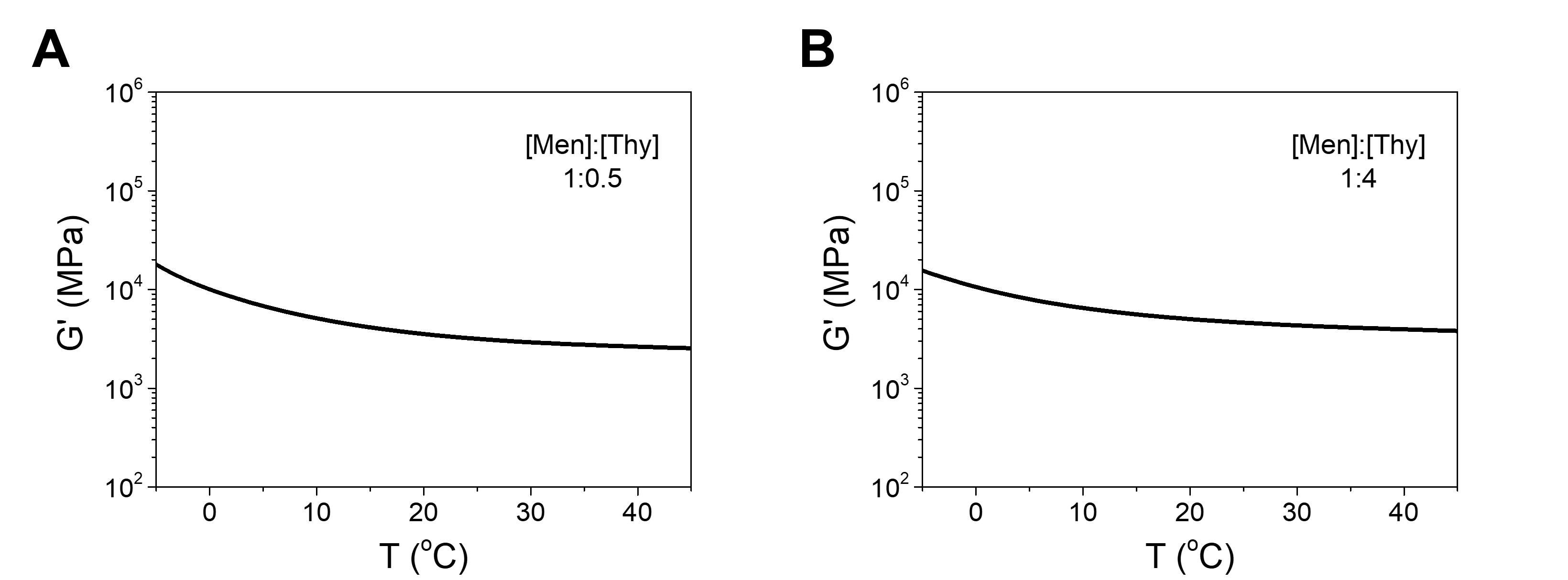


**Figure S10**. Storage moduli of PBnA eutectogels prepared in DES with [Men]:[Thy] molar ratios of 1:0.5 (A) and 1:4 (B) measured by rhology.


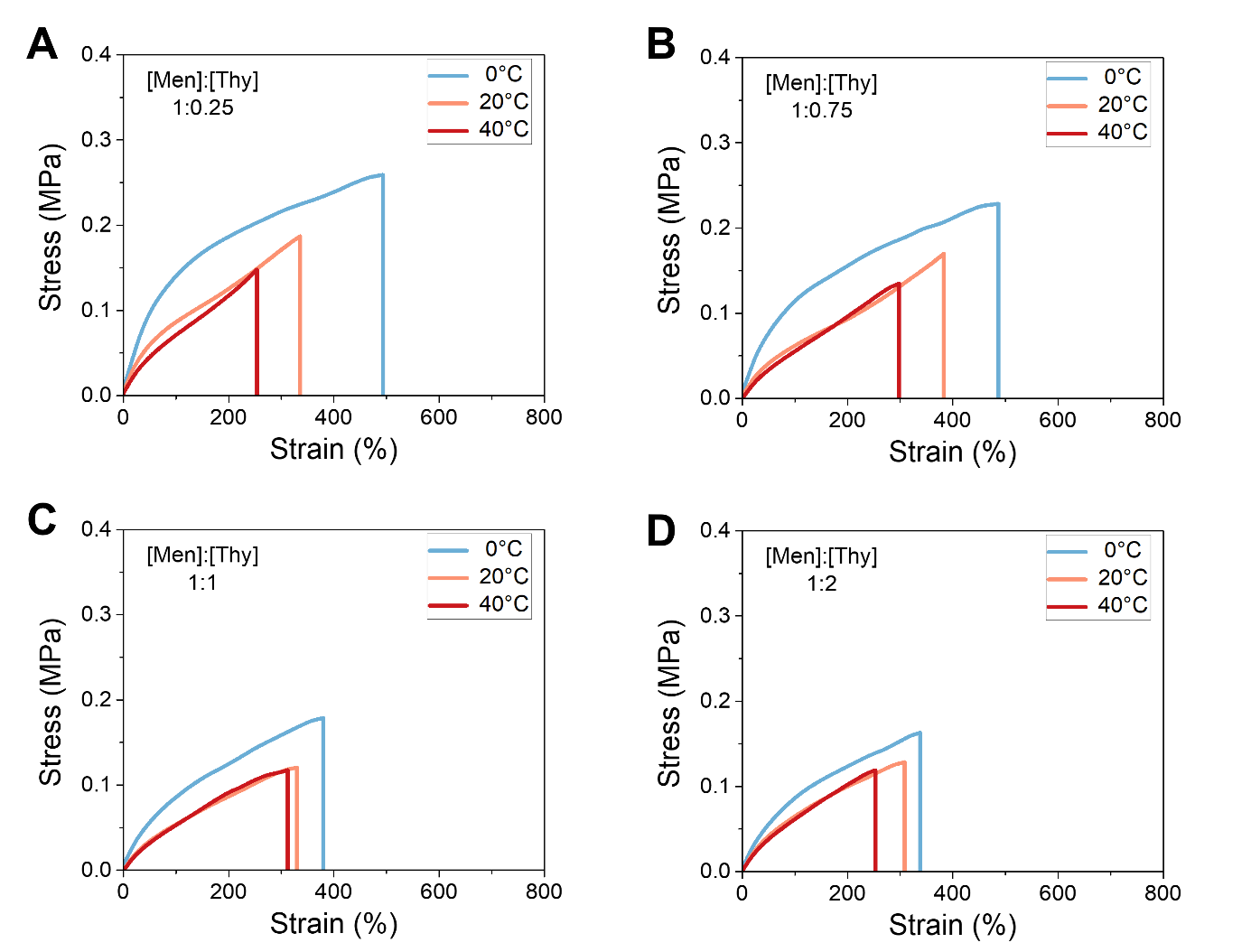


**Figure S11**. Tensile stress-strain curves of PBnA eutectogel prepared in DES with a [Men]:[Thy] molar ratio of 1:0.25 (A), 1:0.75 (B), 1:1 (C), and 1:2 (D).


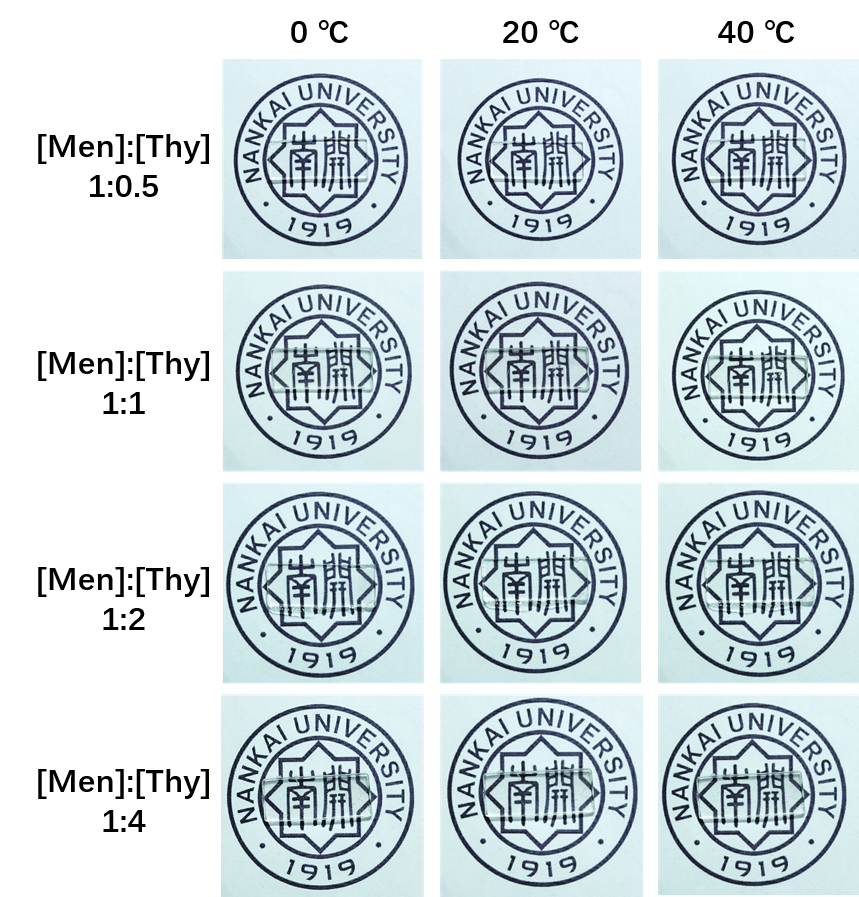


**Figure S12**. Photographs of poly(butyl acrylate) eutectogels at various temperatures. The gels were prepared in menthol:thymol DESs with various [Men]:[Thy] molar ratios as labelled. The crosslinker was EGDMA.


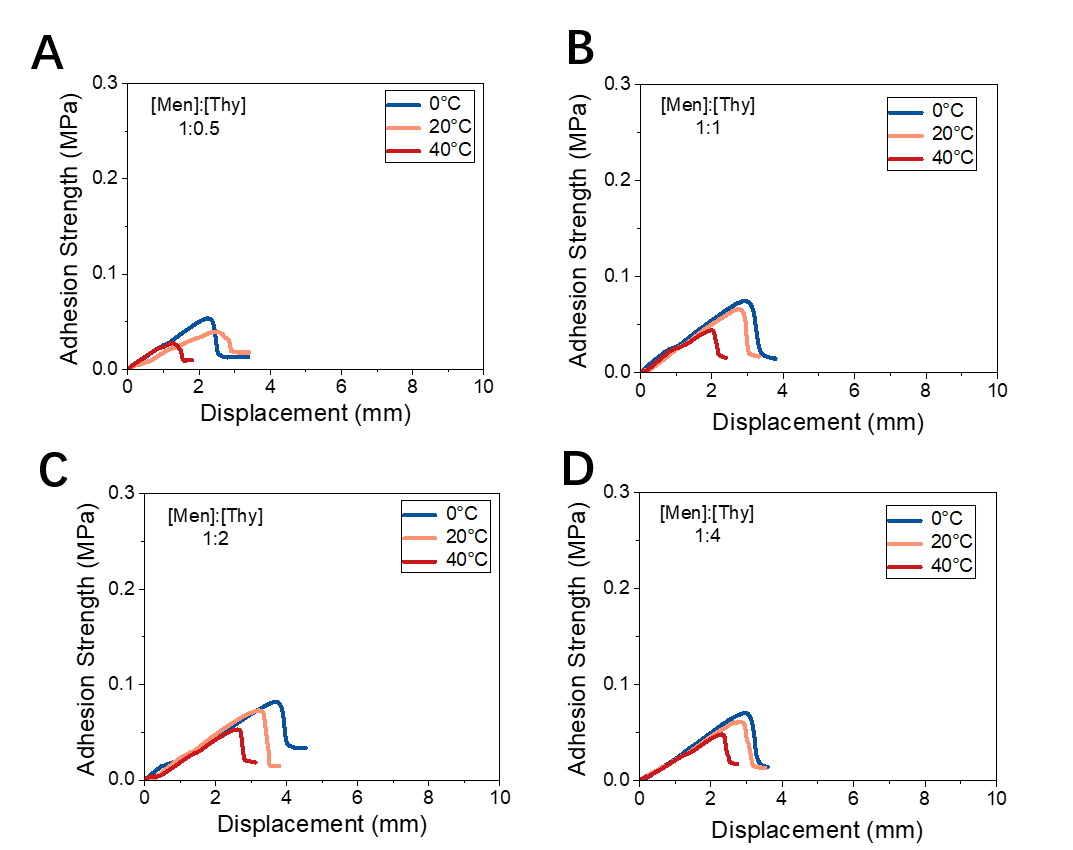


**Figure S13**. Shear peel curves of poly(butyl acrylate) eutectogels prepared in DES with a [Mem]:[Thy] molar ratio of 1:0.5 (A), 1:1 (B), 1:2 (C), and 1:4 (D) measured at 0 °C, 20 °C and 40 °C.

**Figure S14**. Switching ratio (the ratio of adhesion strength at 0°C and adhesion strength at 40°C) of poly(butyl acrylate) gel in menthol:thymol DESs with various [Men]:[Thy] molar ratios.


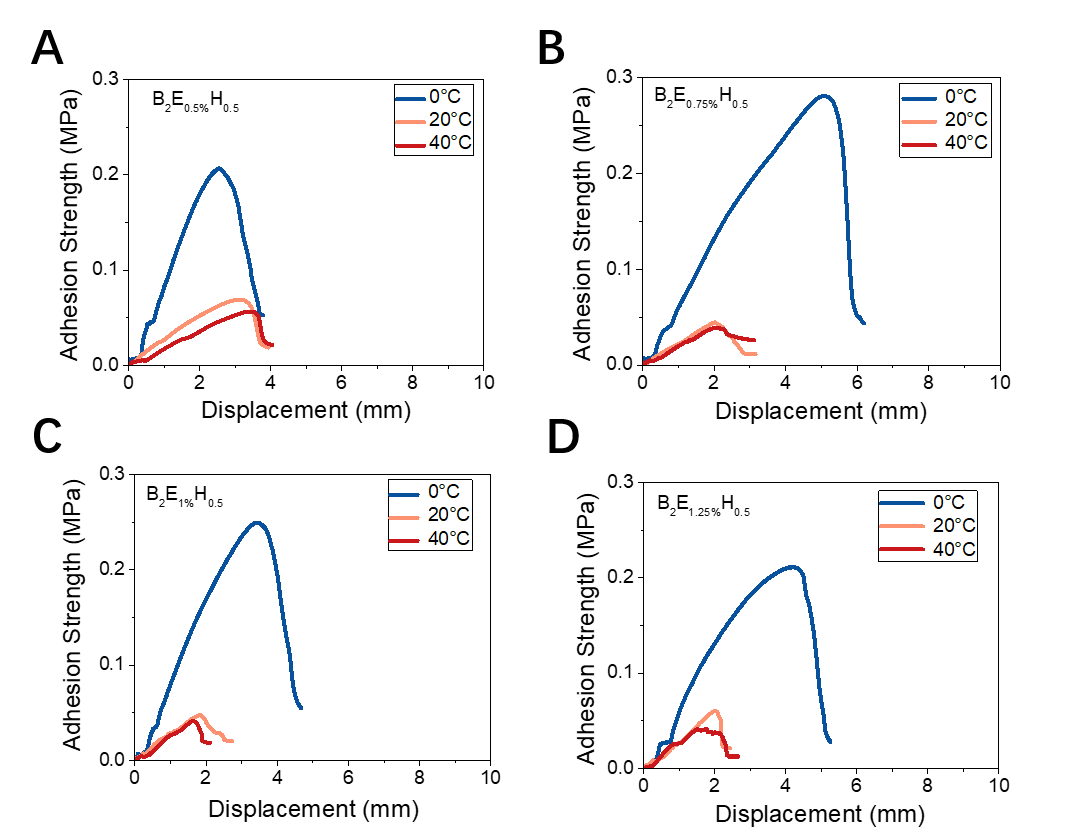


**Figure S15**. Shear peel curves of PBnA eutectogels measured at 0 °C, 20 °C and 40 °C. The gels were prepared using a BnA content of 2 g, a DES content of 0.5, and an EDGMA content of 0.5% (A), 0.75% (B), 1% (C), and 1.25% (D).

**Figure S16**. Switching ratio (the ratio of adhesion strength at 0°C and adhesion strength at 40°C) of PBnA eutectogels with different EGDMA contents.


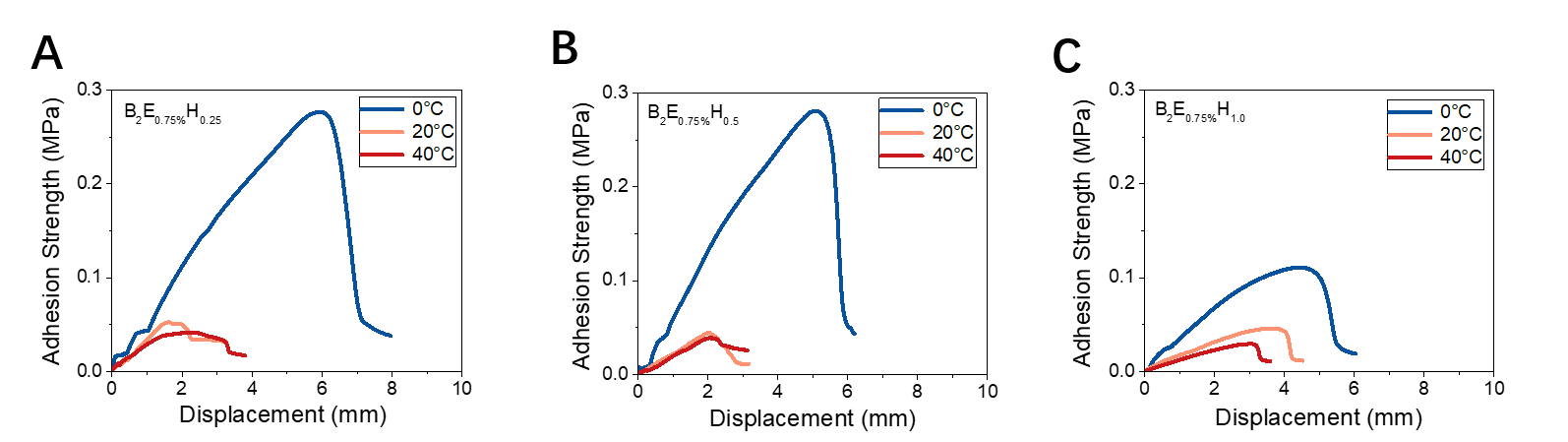


**Figure S17**. Shear peel curves of PBnA eutectogels measured at 0 °C, 20 °C and 40 °C. The gels were prepared using a BnA content of 2 g, an EDGMA content of 0.5%, and a DES content of 0.25 (A), 0.5 (B), and 1.0 (C).

**Figure S18**. Switching ratio (the ratio of adhesion strength at 0°C and adhesion strength at 40°C) of PBnA eutectogels with different DES contents.


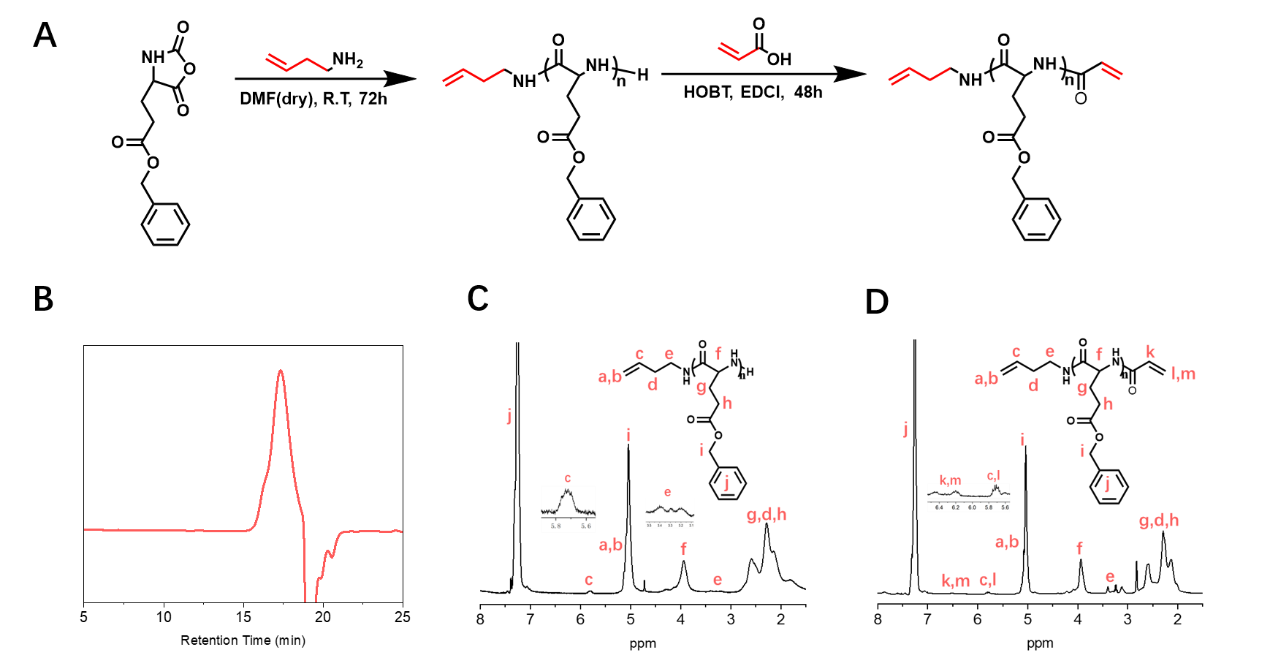


**Figure S19**. (A) Synthetic route of the PBLG-based peptide crosslinker. （B）GPC trace of PBLG precursor in DMF solution. (C, D) ^1^H NMR spectra of PBLG precursor (C) and the final product (D). The solvent is CDCl_3_.


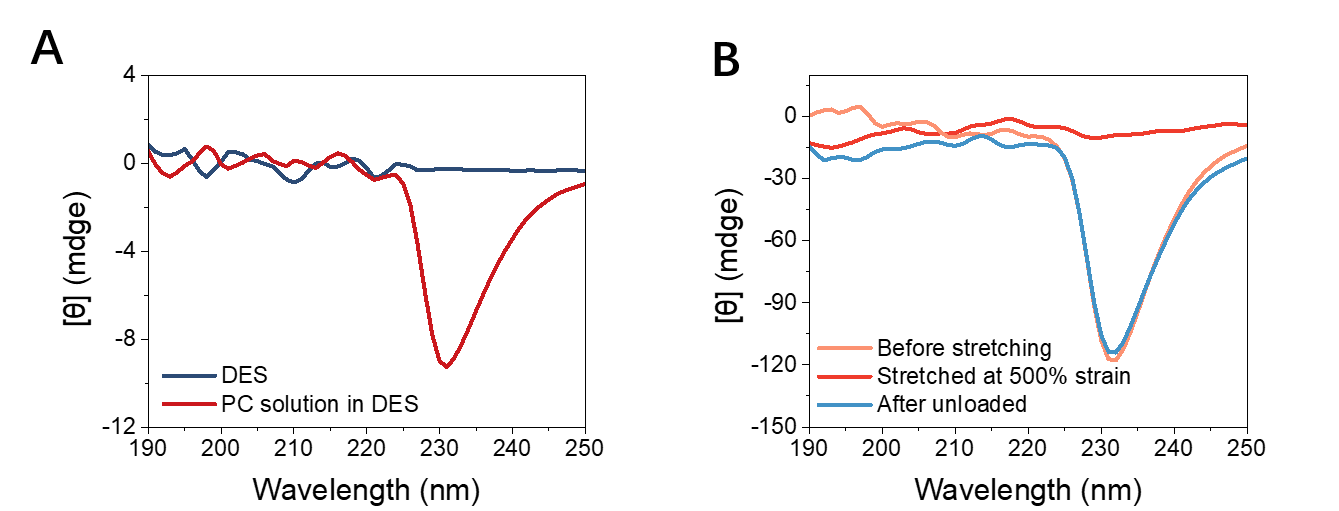


**Figure S20**. (A) CD spectra of menthol:thymol DES and P22 solution in DES. (B) CD spectra of a B_2_PC_0.75%_H_0.5_ eutectogel before stretching, stretched at 500% strain and after unloaded.


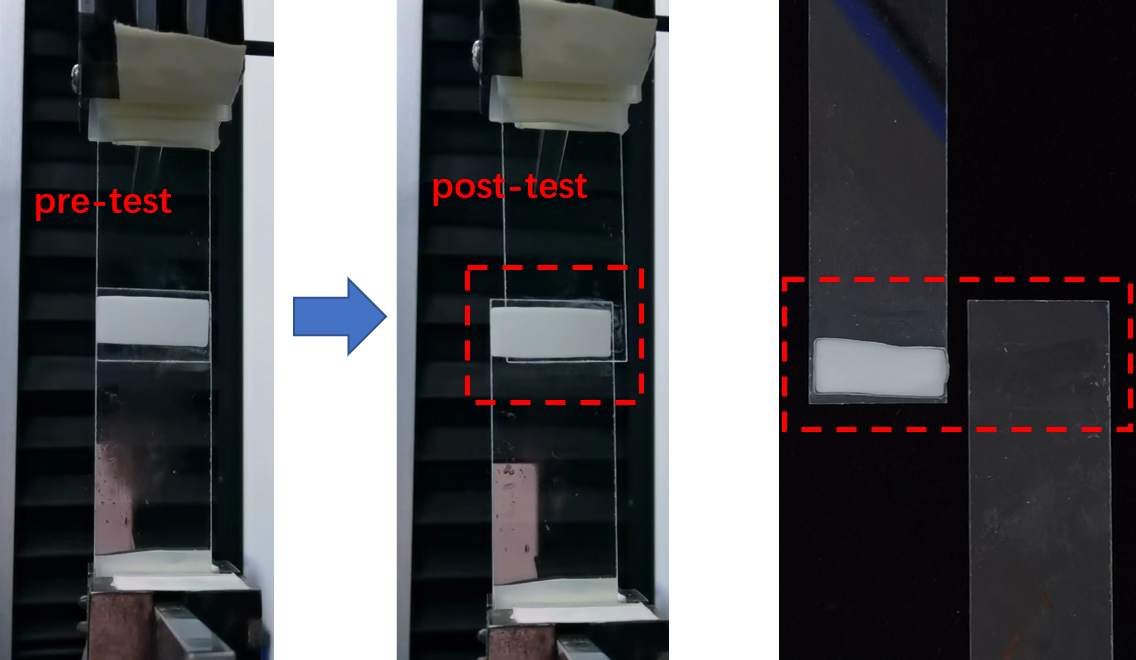


**Figure S21**. Photographs of a B_2_PC_0.75%_H_0.5_ gel before and after lap shear test. After detachment, the gel remained intact and no residue was left on the substrate.


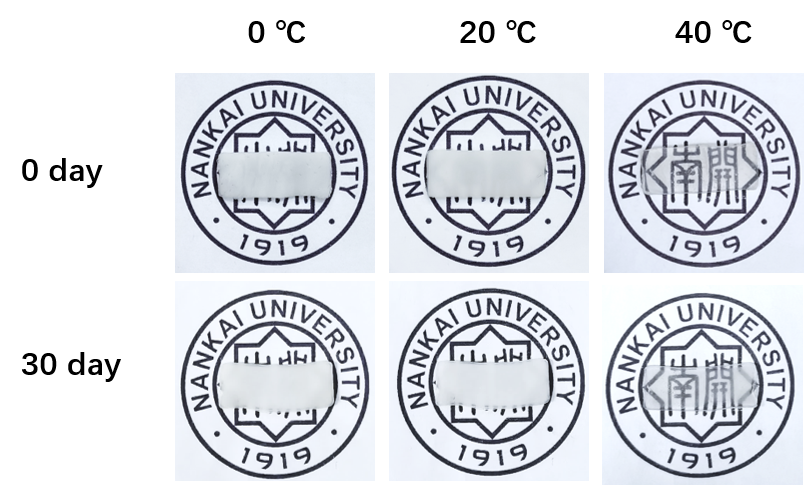


**Figure S22**. Photographs of a B_2_PC_0.75%_H_0.5_ gel before and after 30 days of storage under ambient conditions. The photographs were taken at various temperatures.

**Figure S23**. Weight change of a B_2_PC_0.75%_H_0.5_ eutectogel stored in a humid environment for 30 days.

**Figure S24**. UCST of a B_2_PC_0.75%_H_0.5_ eutectogel before and after 30 days of storage under ambient conditions.


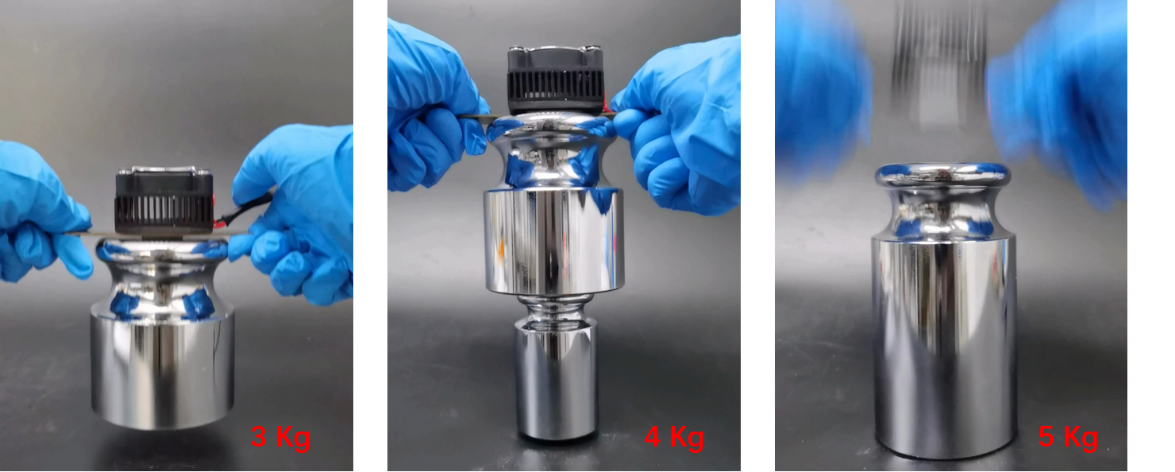


**Figure S25**. Lifting weights with different masses using the B_2_PC_0.75%_H_0.5_ gel-based gripper. The 3kg and 4kg weights were successfully lifted, but the 5 kg weight was not.

**Table S1.** Synthesis and characterization of poly(benzyl acrylate) by RAFT polymerization.

| Sample | [M]/[I] | Mw | Mw/Mn | DP |
| --- | --- | --- | --- | --- |
| PBnA_50_ | 50/1 | 1.1×10^5^ | 1.19 | 42 |
| PBnA_100_ | 100/1 | 2.3×10^5^ | 1.22 | 87 |
| PBnA_150_ | 150/1 | 3.5×10^5^ | 1.23 | 134 |
| PBnA_200_ | 200/1 | 4.9×10^5^ | 1.23 | 187 |

**Table S2**. Synthesis and characterization of peptide crosslinker.

| **Name** | **Monomer/Initiator** | **Mn** | **Mw** | **Mw/Mn** | **DP** |
| --- | --- | --- | --- | --- | --- |
| PC | 25 | 4.2×10^3^ | 4.8×10^3^ | 1.12 | 22.46 |

**Table S3**. Comparison of the reversible adhesive gel with the previously reported materials.

| **Adhesives** | **Materials** | **High adhesion** | **low adhesion** | **External stimulus** | **Mechanism** | **Versatility** |
| --- | --- | --- | --- | --- | --- | --- |
| **This work** | **eutectogel** | **627 kPa** | **45 kPa** | **Temperature** | **Peptide enhancement and Phase separation** | **Glass, Steel, Copper, PTFE, PMMA, PP** |
| *[1]* | Hydrogel | 31.25 kPa | 0.13 kPa | Temperature | Microscale and mesoscale contact coupling changes | Wood, Ti, Fe, Al, Glass, Si, PET |
| *[2]* | Hydrogel | 11.5 kPa | 0.5 kPa | electric | Screening of the catechol groups | Hydrophilic/Hydrophobic Glass, Ceramic, Copper, PMMA, PTFE |
| *[3]* | Hydrogel | 6.31 kPa | 1.343 kPa | pH | Screening of the catechol groups | quartz |
| *[4]* | Hydrogel | 20 kPa | 5 kPa | electricity | Molecular rearrangement | (bovine) aorta, lung, cartilage, cornea |
| *[5]* | Hydrogel | 18 kPa | 5 kPa | pH | Screening of the catechol groups | SiO_2_ |
| *[6]* | Hydrogel | 400 J/m^2^ | 50 J/m^2^ | solvent | Reversible covalent crosslinking | Biological tissue |
| *[7]* | Hydrogel | 200 J/m^2^ | 10 J/m^2^ | UV | Phase transition | Elastomers, Inorganic solid, Porcine, Liver, Skin |
| *[8]* | Hydrogel | 37.4 kPa | 6.8 kPa | temperature | Phase transition | PE, Al, Steel, PTEF, Glass, PP, Rubber, Porcine skin |
| *[9]* | Hydrogel | 3.18 kPa | 0.63 kPa | Temperature | Sol–gel transition | Glass, Pork tissue, PVA hydrogel, Pork skin |
| *[10]* | Polymer | 8.1 kPa | 1.8 kPa | NIR | Screening of the  catechol groups | Al, Si, PP, PS, PET, PTFE, PI, Glass |
| *[11]* | Polymer | 4.0 kPa | 2.0 kPa | Temperature | Screening of the catechol groups | Al, Si, Ti, PTFE, Glass |
| *[12]* | Hydrogel | 6.2 kPa | 1.8 kPa | Temperature | Screening of the catechol groups | Chicken meat, Metal, Eggshell, Teflon septum |

**References**:

[1] Z. Zhang, C. Qin, H. Feng, Y. Xiang, B. Yu, X. Pei, Y. Ma, F. Zhou, *Nat. Commun.* **2022**, *13*, 6964.

[2] J. Huang, Y. Liu, Y. Yang, Z. Zhou, J. Mao, T. Wu, J. Liu, Q. Cai, C. Peng, Y. Xu, B. Zeng, W. Luo, G. Chen, C. Yuan, L. Dai, *Sci. Robot.* **2021**, *6*, eabe1858.

[3] A. R. Narkar, B. P. Lee, *Langmuir* **2018**, *34*, 9410.

[4] L. K. Borden, A. Gargava, S. R. Raghavan, *Nat. Commun.* **2021**, *12*, 4419.

[5] A. R. Narkar, C. Kendrick, K. Bellur, T. Leftwich, Z. Zhang, B. P. Lee, *Soft Matter* **2019**, *15*, 5474.

[6] X. Chen, H. Yuk, J. Wu, C. S. Nabzdyk, X. Zhao, *PNAS* **2020**, *117*, 15497.

[7] Y. Gao, K. Wu, Z. Suo, *Adv. Mater.* **2019**, *31*, 1806948.

[8] X. Shi, P. Wu, *Small* **2021**, *17*, 2101220.

[9] R. Xu, S. Ma, Y. Wu, H. Lee, F. Zhou, W. Liu, *Biomater. Sci.* **2019**, *7*, 3599.

[10] Y. Ma, S. Ma, Y. Wu, X. Pei, S. N. Gorb, Z. Wang, W. Liu, F. Zhou, *Adv. Mater.* **2018**, *30*, 1801595.

[11] Y. Zhao, Y. Wu, L. Wang, M. Zhang, X. Chen, M. Liu, J. Fan, J. Liu, F. Zhou, Z. Wang, *Nat. Commun.* **2017**, *8*, 2218.

[12] A. Eklund, O. Ikkala, H. Zhang, *Adv. Funct. Mater.* **2023**, *n/a*, 2214091.

**Captions for movies:**

**Movie S1**: Two pieces of stainless steel were bonded together with a B_2_PC_0.75%_H_0.5_ gel and cooled to 0 °C. The device did not debonded when put on a 12 Kg bucket, but failed when heated with a hair dryer.

**Movie S2**: Lifting a 1 kg weight using the B_2_PC_0.75%_H_0.5_ gel-based gripper at 40^o^C..

**Movie S3**: Lifting a 1 kg weight using the B_2_PC_0.75%_H_0.5_ gel-based gripper at 0^o^C.

**Movie S4**: Grasping, transporting and releasing a 1kg weight using the B_2_PC_0.75%_H_0.5_ gel-based gripper.

**Movie S5**: The robot climbs on vertical copper surface.

**Movie S6**: The robot climbs on inverted copper surface.
